# Education in Health Research Methodology: Use of a Wiki for Knowledge Translation

**DOI:** 10.1371/journal.pone.0064922

**Published:** 2013-05-31

**Authors:** Michele P. Hamm, Terry P. Klassen, Shannon D. Scott, David Moher, Lisa Hartling

**Affiliations:** 1 Alberta Research Centre for Health Evidence, Department of Pediatrics, Faculty of Medicine and Dentistry, University of Alberta, Edmonton, Canada; 2 Manitoba Institute of Child Health and Department of Pediatrics and Child Health, Faculty of Medicine, University of Manitoba, Winnipeg, Canada; 3 Department of Pediatrics, Faculty of Medicine and Dentistry, University of Alberta, Edmonton, Canada; 4 Faculty of Nursing, University of Alberta, Edmonton, Canada; 5 Ottawa Hospital Research Institute, Ottawa, Canada; Universidad Peruana Cayetano Heredia, Peru

## Abstract

**Introduction:**

A research-practice gap exists between what is known about conducting methodologically rigorous randomized controlled trials (RCTs) and what is done. Evidence consistently shows that pediatric RCTs are susceptible to high risk of bias; therefore novel methods of influencing the design and conduct of trials are required. The objective of this study was to develop and pilot test a wiki designed to educate pediatric trialists and trainees in the principles involved in minimizing risk of bias in RCTs. The focus was on preliminary usability testing of the wiki.

**Methods:**

The wiki was developed through adaptation of existing knowledge translation strategies and through tailoring the site to the identified needs of the end-users. The wiki was evaluated for usability and user preferences regarding the content and formatting. Semi-structured interviews were conducted with 15 trialists and systematic reviewers, representing varying levels of experience with risk of bias or the conduct of trials. Data were analyzed using content analysis.

**Results:**

Participants found the wiki to be well organized, easy to use, and straightforward to navigate. Suggestions for improvement tended to focus on clarification of the text or on esthetics, rather than on the content or format. Participants liked the additional features of the site that were supplementary to the text, such as the interactive examples, and the components that focused on practical applications, adding relevance to the theory presented. While the site could be used by both trialists and systematic reviewers, the lack of a clearly defined target audience caused some confusion among participants.

**Conclusions:**

Participants were supportive of using a wiki as a novel educational tool. The results of this pilot test will be used to refine the risk of bias wiki, which holds promise as a knowledge translation intervention for education in medical research methodology.

## Introduction

Knowledge translation (KT) strategies for delivering education and professional development to health care providers are of great interest in optimizing health services and delivery. The Effective Practice and Organisation of Care (EPOC) Review Group within the Cochrane Collaboration has been particularly instrumental in synthesizing the evidence and evaluating the effectiveness of interventions aimed at improving the delivery, practice, and organization of health services, including in continuing education and quality assurance [1]. Traditional KT interventions that have demonstrated effectiveness in EPOC systematic reviews include printed educational materials (4.3% absolute improvement in categorical process outcomes versus no intervention) [2], combining didactic and interactive content in the distribution of educational materials (13.6 median adjusted risk difference (RD) in outcomes for professional practice versus didactic (RD 6.9) or interactive (RD 3.0) sessions alone) [3], and endorsement by local opinion leaders (12% median absolute increase in compliance in behaviour versus no intervention, an alternative intervention, or multiple alternative interventions) [4]. While extensive research has been conducted with respect to changing clinician behaviour, the impact of KT strategies on researchers’ behaviour has not been explored to date.

There is a body of evidence suggesting that pediatric randomized controlled trials (RCTs) are susceptible to methodological limitations, and a substantial proportion of the studies conducted are at a high risk of bias [5]–[14], increasing the likelihood that treatment effects are being exaggerated. In two evaluations that assessed the overall risk of bias of pediatric RCTs using the Cochrane Collaboration’s Risk of Bias tool, more than 90% of studies were at high or unclear risk of bias, and these trials reported larger effect estimates than studies at low risk of bias [12], [14]. Guidance on rigorous trial conduct and reporting is available in abundance, demonstrating the negative impact of design elements such as improper sequence generation, allocation concealment, and blinding [15]–[25]; as is research on specific challenges inherent to trials in child health, for example, recruitment and consent [26]–[30]. However, a research-practice gap persists between what is known about bias and how RCTs are conducted, indicating a need for KT research in this population.

In previous work investigating the barriers and facilitators to the uptake of methodological principles in child health research, pediatric trialists indicated that a lack of formal training in research methods and a negative research culture adversely impacted their ability to conduct RCTs to the highest standards, while contact with knowledgeable and supportive colleagues had a beneficial effect [31]. In this context, we endeavored to develop a KT intervention for researchers that would be tailored to address these factors [32].

Social media tools have recently begun to be explored as KT interventions in educational contexts [33]. Wikis, collaborative websites that can be edited by all users [34], provide a unique opportunity to build on existing KT research and to be used as novel tools in disseminating information. Due to the flexibility in their formatting, wikis can be created to incorporate a number of successful elements of other strategies, such as interactivity alongside static educational content and involvement of opinion leaders. Additionally, a wiki could act as a centralized resource centre for materials related to trial methodology, while promoting a positive research culture and providing a supportive online community, in response to the key factors identified by researchers in the field. There are a few existing models of wikis that have been used to disseminate methods research, including sites dedicated to ethical issues related to the conduct of cluster randomized trials [35] and knowledge translation terminology [36], but their use is not yet widespread. Both of these wikis are used largely to post content, but are also intended to facilitate discussion and solicit user feedback.

Given the consistency and extent of the evidence suggesting that the methodological rigor of pediatric RCTs must be improved, research must now turn to methods of ensuring the pediatric trialists are in a position to conduct high quality research. To address this agenda, the objective of this study was to develop and pilot test a wiki designed to educate child health trialists and trainees in the principles involved in minimizing risk of bias in RCTs. The primary focus of this work was to conduct a preliminary evaluation of the usability of the wiki using qualitative methods; future research will focus on the effectiveness of the wiki as a knowledge translation intervention.

## Methods

### Wiki Development

The wiki was developed using Wikispaces, a free host platform [37]. In order to maximize credibility and familiarity, the wiki was established under the auspices of StaR Child Health, an international initiative dedicated to improving the quality of pediatric clinical research [38], and is available at www.starchildhealth-riskofbias.wikispaces.com. Specifically, the wiki was designed to contribute to the KT agenda of the StaR Child Health Risk of Bias Standard Development Group [39]. Content was structured to emphasize two main areas: risk of bias and the conduct of pediatric RCTs. Guidance on minimizing risk of bias followed the framework developed by the Cochrane Collaboration and was focused on seven key domains: sequence generation, allocation concealment, blinding of participants and personnel, blinding of outcome assessors, incomplete outcome data, selective outcome reporting, and other sources of bias [40]. Overall, the wiki comprises six major sections: an introduction, a page for each of the risk of bias domains including resources and interactive examples, issues specifically relevant to pediatric trials, a discussion forum, tools, and references.

Development of the wiki followed the three main steps of tailoring interventions: 1) identification of the barriers and facilitators faced by the target users; 2) matching the intervention to the identified factors; and 3) applying and assessing the tailored intervention [41]. A theoretical foundation was also applied, drawing on Diffusion of Innovations [42] to outline the key attributes of innovative ideas or technologies (relative advantage, compatibility, complexity, trialability, observability) and focus theory of normative conduct [43], which states that motivation for behavioural change can arise through emphasis on what ought to be done (i.e., encouraging rigorous trial conduct) versus what is done (i.e., highlighting the prevalence of poorly conducted trials). Diffusion of Innovations was used to focus the selection of a wiki as the KT intervention. A wiki was felt to confer a relative advantage over other resources as it would compile materials into one location; online learning would be compatible with the lives of busy health care professionals; wikis are user-friendly, minimizing the complexity required to adopt the innovation; and the ability for users to benefit from the content of the site without being required to participate would allow for both trialability and observability. The focus theory of normative conduct was employed to guide the structure of the wiki content in terms of framing the message in a manner that would maximize the likelihood of its uptake. Several KT strategies were incorporated into the wiki design, interactivity prominent among them. Much of the educational content is intended to be static, but this appears alongside interactive components, including examples that users can work through, discussion forums, editing capabilities, and social media (Twitter feed). The wiki has been endorsed and promoted by key members of StaR Child Health, who are recognized leaders in the fields of pediatrics and trial methodology. Technological elements and formatting of the wiki were informed by the U.S. Department of Health and Human Services’ (HHS) guidelines on web design and usability [44].

### Target Audience and Recruitment

The target audience for the wiki pilot test consisted of both clinical trialists, to ensure relevance of content, and methodologists, to ensure accuracy. We were interested in conducting a preliminary evaluation of the content and format of the wiki; therefore, we endeavored to maintain a diverse range of perspectives from participants to represent the broad spectrum of backgrounds and experience levels of researchers learning about RCT methodology. Participants were recruited from three sampling frames: students enrolled in systematic review and randomized controlled trials courses, pediatric trialists, and methodologists affiliated with the Agency for Healthcare Research and Quality Evidence-based Practice Center Program. Recruitment occurred between March and June 2012 through presentations to groups of trainees, promotion at prominent pediatric and methodological conferences, and targeted email requests.

### Ethics Statement

This study was approved by the Health Ethics Research Board at the University of Alberta.

### Data Collection

To evaluate the usability of the wiki, we conducted semi-structured interviews that were based on the major constructs identified in the HHS guidelines: user perceptions of consistency, efficiency, productivity, organization, ease of use, intuitiveness, and straightforwardness [44]. User preferences regarding content and formatting were ascertained to inform the modification of the prototype version of the wiki. We aimed to conduct approximately 12 interviews to reach saturation, the point at which no new information emerges from the data. Our operational definition for reaching saturation was when no new major themes were identified from three consecutive interviews. Each participant signed and returned a consent form prior to the conduct of the interview. Interviews were 30 to 60 minutes and conducted in person or by telephone by the first author (MPH). All interviews were recorded and transcribed verbatim.

To complement the qualitative data, quantitative measures of web traffic were collected to explore how the site was being used. Data were collected both through the usage statistics built into the Wikispaces platform, and through Google Analytics. Measures included number of unique visitors, geographic location, and page views.

### Data Analysis

We used directed content analysis to code interviews [45], identifying categories in the data that described usability, user preferences, and feedback for modification and improvement (Table S1). The lead author coded the data in consultation with the rest of the study team. We conducted qualitative data collection and analysis concurrently, following an iterative process [46]. We used NVivo to manage qualitative data (www.qsrinternational.com). As supplementary, contextual information, quantitative data is presented descriptively, using frequencies and proportions.

## Results

**Figure 1:**
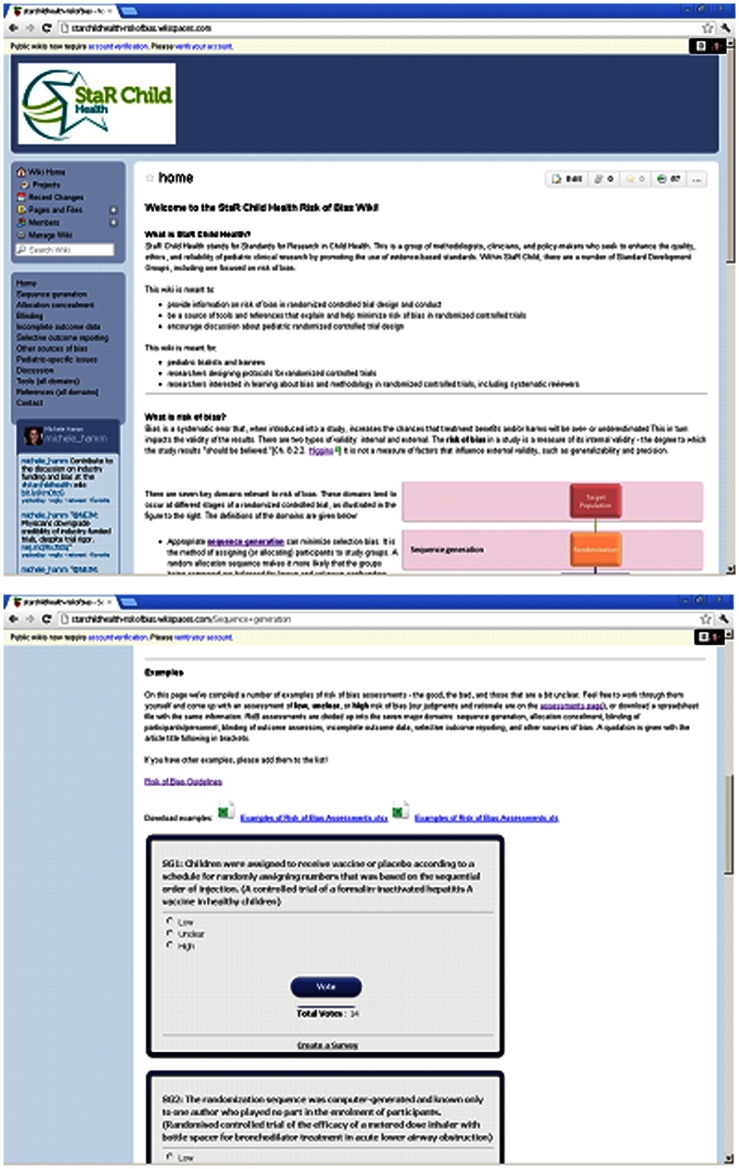
Screen shots of the home page and examples included in the wiki.

The wiki was pilot tested with 15 participants, at which point saturation was reached. Four were trained as physicians, six were PhD-trained researchers, four were PhD students, four were masters-trained researchers (including project coordinators), and one was entering a masters program (research assistant). Six participants specialized in pediatrics, seven had experience conducting RCTs, and nine had experience conducting systematic reviews. Three were new to the concepts of risk of bias. Thirteen were from Canada, one was from the United Kingdom, and one was from the Netherlands. Results were similar between trialists, methodologists, and trainees. Screen shots of the wiki are included in Figure 1.

### Usability

All of the participants found the wiki to be well organized, easy to use, and straightforward to navigate (Table 1). The simplicity of the site was seen as a strength, and it was found to be logical and user-friendly. Respondents liked the layering of the wiki, with its focus on general and introductory content, with links and references to more detailed or complex information. The content and language was easy to understand, with only minor suggestions for clarification. While much of the background information included in the wiki is available through other sources, participants liked that the site provided a centralized collection of this content, making it easier to find and work through.

**Table 1:** Typical comments on the usability of the wiki.

*07 (trialist, systematic reviewer)* – I thought everything was really easy to read and easy to follow and not too scientific, like I could follow everything.
*11 (trialist)* – Navigation was quite easy. So I did find myself, you know, you’d be reading from the home page and then click in to get to more information, and then I’d click on something else – so I’d get myself off track and off the home page, but that was just my own personality, or the way I navigate a page, but it was nice to be able to go deeper and deeper. It did take me away from the text, but I was always able to get back to the home page quite easily, so that was good.
*11 (trialist)* – I think that if people wanted the quicker view, you could stick to the home page, and then you know, if you find the details excessive, then people don’t have to click into the extra text in each side heading. So no, I think that the way it was organized gave either a brief overview or more in-depth – I think the choice was useful.
*09 (trialist, systematic reviewer)* – It did strike me as a nice centralized place to have all that information. Most of it, as far as I can tell, is out there somewhere; the question is finding it all in one place. I thought that was good there.

### User Preferences

Participants liked the additional features of the site that were supplementary to the text and wanted to see more added in (Table 2). In particular, they liked case studies and real world illustrations, interactive polls that served as teaching examples, diagrams, and the Twitter feed. The polls were structured to provide an excerpt from a published trial and the user could assess the example as being at low, unclear, or high risk of bias. Their responses would then be presented along with those of other users. Although we deliberately did not include a ‘correct’ answer due to the inherent subjectivity of the assessments, many participants felt that this would have been helpful. One respondent felt that allowing for voting took away from the credibility of the site.

**Table 2:** Typical comments on user preferences.

Supplementary features
*16 (psychologist)* – I clicked on a couple of the links to the Twitter leads as well. And that’s quite nice, because that gives sort of a current flavor, […] real-world things that people are talking about.
*03 (project coordinator)* – I really like this [case study from a medical drama storyline]. This was, for me, tied in to pop culture – like what are most people familiar with? You could relate to it, and it’s a clear example right there. So it’s not just a whole lot of theory. And I think that’s why the polls were kind of nice as well.
*16 (psychologist)* – … real life examples that happen quite commonly in clinical trials. I think that’s quite nice. It’s one thing to learn about in the abstract, but when you know that it’s happened in the real world, it’s a real thing that can happen and that you need to watch out for this, I find that’s quite powerful.
**Example polls**
*03 (project coordinator)* – [The examples were] a really interactive way for people to actually sit down, like for me not to know a lot about risk of bias, and be learning it on the go, and you don’t have to do a lot to learn piece by piece. Like if I had to go do something else, then I could still go back to it and pick away at it, […] then you don’t feel like it’s too intensive.
*01 (trialist, systematic reviewer)* – But does it tell you whether you’re right or wrong in the end?
*14 (trialist, systematic reviewer)* – I answered the poll, and then you sort of see how other people have answered the poll. But maybe that’s just me being sort of scholastic, but I’d be sort of curious about what the answer is. But I don’t think there is an answer, is there? And then the more I thought about it, I think that’s the point, is that you know, two really smart people could answer the same question different ways, and not necessarily one of them is right or wrong.
*10 (trialist)* – To see votes, I mean it’s like a beauty contest or something like that […] and it’s not very attractive, I think, to people who want to do serious scientific work.
**Technological features**
*13 (research assistant)* – To me, it wouldn’t be as big of a deal to have [the text of the examples separated from the polls in a less esthetically pleasing format] than to have the page slowed down [by large text boxes].
*14 (trialist, systematic reviewer)* – I noticed that it was slow every time I was on it.
*12 (systematic reviewer)* – I like the fact that everything – it’s not overwhelming. I’m not much for websites with lots of bells and whistles, so I like the fact that it’s not overwhelming, but it’s not bland either.
*16 (psychologist)* – I think one of its beauties is its simplicity.
*13 (research assistant)* – I thought it would be cool […] if you could have the [risk of bias] guidelines move along down the page [beside the examples while scrolling].
*01 (trialist, systematic reviewer)* – I know this is an over-simplification of the whole process, but people tend to like wizards. Where you’re asked a question and you say for example, “was this study randomized?” Or “will this study be randomized?” And then they say yes or no, and then based on that, you get a second question and so on. And at the end they would get their answer.

While users liked the example polls, they found that they caused the pages to load slowly, which was a significant disadvantage (Table 2). There was an interesting contrast in the comments between wanting to maintain the simplicity of the site and suggesting the addition of more technologically advanced features, such as in the presentation of the examples, the use of tabs for navigation, and creating links within figures. This difference tended to run across generational lines, with younger participants more at ease with a wider range of digital functionality.

### Audience

Given the use of the Cochrane Collaboration’s framework for risk of bias to structure the wiki, there was some uncertainty regarding the intended audience, specifically whether it was targeted for trialists or systematic reviewers and whether the connection between assessing risk of bias in a published study and addressing it in the design and conduct of a trial would be apparent.

“I think it has to be clear somewhere […] that risk of bias is not a guideline for conducting a trial; it’s just highlighting some elements that will enhance the quality, internal validity of the study, and so on.” –*08 (systematic reviewer).*

Suggestions were made to add more tools that focused on the pragmatic issues related to conducting an RCT to increase the site’s relevance to trialists. Resources on the wiki such as tips on how to blind surgical trials were viewed as being useful, and participants wanted to see more tools like these. Other comments were focused on tailoring information to different user groups. With potential applications for trialists and systematic reviewers or methodologists, participants suggested that it could be useful to either divide content into sections that would be most relevant to different groups, or to provide a framework up-front explaining how different users should make use of the site.

### Web Traffic

Over the study period (May 3– July 5, 2012), 240 unique visitors accessed the wiki. Nearly all visits were from Canada (87.6%), followed by the United Kingdom (2.7%), the United States (2.4%), and the Netherlands (1.8%). After the home page, the most highly accessed pages within the wiki were the domain-specific pages for sequence generation (2^nd^) and allocation concealment (3^rd^), and the comprehensive tools page (4^th^), compiling the tools and resources for all of the risk of bias domains. The pages that were intended to encourage interactivity, namely the pediatric-specific issues and discussion pages, were accessed 11^th^ and 12^th^ most frequently, respectively, out of 40 pages. While not a specific focus of this pilot test, it is noteworthy that there were no contributions to any of the discussion forums. With an emphasis on the content and formatting of the wiki, and not explicitly on the use of the interactive components, the study participants were not required to contribute to any of these forums; however, these results provide exploratory data on how they and other users of the site accessed information. These findings will inform further modifications and evaluations of the wiki.

## Discussion

Overall, the feedback on the risk of bias wiki was positive, with participants viewing this method of dissemination favourably. Suggestions for improvement were largely related to issues of clarification or esthetics, rather than the content, format, or usability. Participants were interested in the opportunities provided by the wiki as a relatively novel educational tool, and felt that this platform held potential for future uses in providing methodological training.

One of the concerns related to using a wiki in the educational realm is that there is no guarantee that the content will be accurate because it can be modified by any wiki user without editorial control [34], [47]–[48]. However, only one respondent in our study voiced this opinion, stating:

“So what I don’t understand is you have this wiki to teach people? I think that’s one of the aims? But at the same time, you allow them to edit what’s in there – isn’t that a bit dangerous? If somebody goes to this wiki and puts in nonsense?” –*10 (trialist).*

This will be an important consideration not only in the authenticity of the wiki content, but also in the site’s sustainability, as it will have implications for the resources required if ongoing monitoring is necessary. A certain level of continued involvement on the part of the developers can be expected, but there is some evidence that online information tends to be self-correcting [49], and with many wiki users preferring to act as passive knowledge consumers, rather than as active editors [50], this may not represent a significant issue.

Obtaining buy-in for the wiki from the target end-users will represent a substantial challenge. Not only are there barriers in terms of encouraging participation once the site has been accessed, but the intended audience of pediatric clinical trialists already faces significant time constraints and is largely part of an environment in which education on research methodology is not highly valued [31]. The use of theory, established KT strategies, and tailoring in the development of the wiki were used to mitigate these obstacles, but do not overcome the challenge of drawing users to the site. The ideal use of a wiki would likely be in the context of a course or training module in which users are motivated or required to participate, in which case it could potentially parallel the successes found in the use of online continuing medical education, where benefits have been found in knowledge gains and in changing clinician behaviour [51], [52]. Additionally, this strategy would align with evidence that multifaceted interventions targeting change are more effective than single interventions [53].

While the KT literature that this study was based on is focused on changing clinician behaviour, it lends itself to adaptation to the target population of trialists, as most are clinician scientists. However, their motivation to change may differ when choosing whether to adopt a new or recommended clinical practice versus a research technique with more subtle or distant benefits. With a lack of available empirical evidence, theory can be used to outline potential strategies to address such challenges. Social influences theories guided our approach to targeting motivation, specifically the contrast between descriptive norms (what is done) and injunctive norms (what ought to be done) emphasized in the focus theory of normative conduct [43]. In future promotion of the wiki, social influence could also play a role by continuing to engage respected opinion leaders. While the motivation of trialists was considered in the design of the wiki, this pilot test did not explicitly measure intention to change as a result of exposure to the intervention. Future research on the effectiveness of the site will include measures of intended behaviour change.

The quality of reporting in trials is an important proxy for the validity of the methods employed, and the advent of reporting guidelines such as the CONSORT Statement (Consolidated Standards of Reporting Trials) [54] has led to the availability of recommendations for standard elements to be reported in any clinical trial. A number of the concepts addressed in the CONSORT checklist overlap with those that are relevant to risk of bias and that are included in the wiki. While the standardization of reporting is a crucial step in ensuring the quality of trial reports, the wiki was designed to be used in a complementary manner further upstream in the research process, at the point of trial design and conduct. The recently published SPIRIT 2013 Statement (Standard Protocol Items: Recommendations for Interventional Trials) [55] similarly addresses this goal.

This prototype wiki was developed specifically as a resource for the Risk of Bias Standard Development Group within StaR Child Health, but it can also potentially serve as a model for resources targeting other key areas in pediatric research, for example, data monitoring committees and recruitment. One of the aims of StaR Child Health is to be at the forefront of guidance for trial design, conduct, and reporting in pediatric research [38], and a series of wiki-based educational resources could contribute to this vision. This pilot study represents the first step in the evaluation of this intervention, and the revised version of the wiki will need to be further evaluated for effectiveness. If shown to be beneficial, an implementation strategy will be devised.

### Limitations

The majority of participants in this study were more experienced in systematic reviews than in RCTs. While this did confer an advantage in that they tended to be familiar with the Cochrane Risk of Bias tool and could provide feedback on the accuracy of the content, there was more feedback related to how to improve the site for reviewers than on what could be useful to trialists. Moving forward, however, the guidance on methodology has been found to be sound, and therefore seeking the input of clinical researchers on relevance can be emphasized in future evaluations.

Nearly all of the comments on the usability of the wiki were positive, raising the question of the possibility of interviewer or response bias. To minimize the likelihood of interviewer bias, a standardized interview guide was used. Further, participants were forthcoming with ideas to improve the layout of the site and for additions that could strengthen the content in the future, suggesting that they did not feel compelled to provide only positive feedback.

There were a number of suggestions for modifications based on incorporating more advanced technology into the wiki. A standard website would allow for more flexibility than a wiki in the inclusion of functions that would streamline the site. However, this would be at the cost of the interactivity that the wiki affords and we felt that a less sophisticated site held more potential as an educational resource due to the user-generated components it supports.

### Conclusions

This pilot study was designed to evaluate the usability of a wiki-based educational resource on methodological rigor in pediatric randomized trials. Participants found the wiki straightforward and easy to use, providing suggestions to improve clarity and esthetics. The interactive format was enticing to users and the components that allowed participation or emphasized practical applications over theory were preferred. Built upon an adaptation of the existing knowledge translation evidence base, the risk of bias wiki holds promise for use as an online educational resource for researchers involved in the conduct and evaluation of trials in child health.

## Supporting Information

Table S1 Interview codebook.
(DOCX)
