# Supplementary material for: Education in Health Research Methodology: Use of a Wiki for Knowledge Translation
Source: PLoS One. 2013 May 31;8(5):e64922. doi: 10.1371/journal.pone.0064922 (PMC3669055; doi:10.1371/journal.pone.0064922)
Supplement: Table S1 — Interview codebook. (DOCX) [file pone.0064922.s001.docx]

**Table S1. Interview codebook**

| **Theme** | **Code** | **Definition** |
| --- | --- | --- |
| Individual pages | Home | Suggested changes for each of the individual pages |
|  | Sequence generation |  |
|  | Allocation concealment |  |
|  | Blinding |  |
|  | Incomplete outcome data |  |
|  | Selective outcome reporting |  |
|  | Other sources of bias |  |
|  | Pediatric-specific issues |  |
|  | Discussion |  |
|  | Tools |  |
|  | References |  |
|  | Examples |  |
| General content | General content | -Content that may go into the tailored sections for different end-users  -General comments that could be incorporated into the wiki content  -Ideas on examples/case studies to incorporate |
|  | Audience | How different user groups might use the wiki (e.g., imagining the time a clinician would have to spend on it) |
| General formatting | General formatting | Appearance, functionality, navigation, polls/other tools that span different domain-specific pages, credibility |
| Preferences | Likes | Participants’ comments on what they liked about the wiki |
|  | Dislikes | Participants’ comments on what they didn’t like about the wiki |
